# Antimicrobial resistance in the Eastern Mediterranean Region: experiences, challenges, and perspectives

**DOI:** 10.3389/fpubh.2025.1655232

**Published:** 2025-09-08

**Authors:** Deema Al Bakri, Maya Joukhadar, Aamer Ikram, Nelea Motriuc, Ghassan M. Matar, Rola Ali Ghanem, Heba Mahrous, Tariq Hassan Taha, Hafiz Ahmad, Priyalatha Muthu, Ibrahim Al Faouri, Haitham Bashier, Magid Al-Gunaid, Mohannad Al Nsour, Yousef Khader

**Affiliations:** ^1^The East Mediterranean Public Health Network, Amman, Jordan; ^2^Diakonie Katastrophenhilfe, Amman, Jordan; ^3^Health Fauji Foundation, Islamabad, Pakistan; ^4^AMR Multi-Stakeholder Partnership Platform, Quadripartite, Rome, Italy; ^5^Department of Experimental Pathology, Immunology, and Microbiology, American University of Beirut, WHO Collaborating Center, Beirut, Lebanon; ^6^CRDF, Amman, Jordan; ^7^World Health Organization Regional Office for the Eastern Mediterranean, Cairo, Egypt; ^8^World Organization for Animal Health Subregional Representation for the Arabian Gulf, Abu Dhabi, United Arab Emirates; ^9^RAK Medical & Health Sciences University, Ras Al-Khaimah, United Arab Emirates; ^10^Polio and Immunization Unite, Public Health Department, The Eastern Mediterranean Public Health Network, Amman, Jordan; ^11^Department of Public Health, Jordan University of Science and Technology, Irbid, Jordan

**Keywords:** antimicrobial resistance (AMR), Eastern Mediterranean Region, national action plans, infection prevention and control, antimicrobial stewardship

## Abstract

Antimicrobial resistance (AMR) is a rapidly growing global health threat that undermines the effectiveness of first-line treatments for serious infectious diseases. During the Eighth Regional Conference of the Eastern Mediterranean Public Health Network (EMPHNET), held in Amman, Jordan, from September 15–18, 2024, this critical issue took center stage under the theme “Advancing Public Health Preparedness and Response.” A dedicated roundtable discussion on AMR explored the challenges of implementing effective AMR surveillance, the widespread misuse of antibiotics, and the urgent need for a One Health approach that integrates human, animal, and environmental health. Speakers emphasized that political commitment, sustainable funding, and cross-sector collaboration are essential to curbing AMR, particularly in the Eastern Mediterranean Region (EMR), where factors such as high antimicrobial consumption, conflict, weak health systems, and poor access to regulated antibiotics exacerbate the problem. The discussion also highlighted the critical roles of laboratories and infection prevention and control (IPC) programs in healthcare settings, both of which are central to AMR surveillance and stewardship. Global efforts, including the WHO’s Global Action Plan on AMR and the AMR Multi-Stakeholder Partnership Platform, were recognized as vital frameworks for fostering international cooperation and guiding regional responses. The roundtable concluded with a call for strengthened governance, enhanced laboratory capacities, improved surveillance systems, scaling up IPC programs and enhanced public awareness campaigns to confront the rising threat of AMR in the EMR.

## Introduction

Antimicrobial resistance (AMR) is a rapidly spreading global health emergency that renders first-line therapies ineffective for treating several serious infectious diseases, including gonorrhea, HIV, tuberculosis, and malaria. According to 2021 estimates, AMR claimed 4.71 million lives worldwide, including 1.14 million attributable to bacterial AMR. By 2050, it is anticipated that the number of deaths attributable to AMR could reach 1.91 million globally, with 8.22 million deaths associated with AMR (1). The majority of these deaths will take place in underdeveloped nations. AMR might have a serious negative impact on the world economy. If AMR is not addressed today, the world’s productivity might decline by up to $100 trillion over the next 35 years, or about $8 trillion less in annual output, by 2050 (2). The global gross domestic product (GDP) may decline by 3.5% (3). Additionally, AMR could push up to 28 million people into poverty by 2050, mainly in low- and middle-income countries (4).

Rising levels of AMR will hinder progress toward many of the Sustainable Development Goals (SDGs), particularly those focusing on health and well-being, poverty reduction, food security, environment, and economic growth (5). While the world has been focused on acute pandemics like COVID-19, AMR has continued to develop, with few interventions to address it at scale. As Tom Frieden noted, “If we use antibiotics when not needed, we may not have them when they are most needed.” (6).

The Eastern Mediterranean Public Health Network (EMPHNET) held its Eighth Regional Conference in Amman, Jordan on the 15th through the 18th of September 2023 in Amman under the theme “Advancing Public Health Preparedness and Response: Challenges, Opportunities, and Ways Forward.” The conference sessions in the form of workshops, forums, and roundtables addressed challenges and identified opportunities to advance public health preparedness and response in the Eastern Mediterranean Region and beyond. The roundtable entitled “Antimicrobial Resistance in the Region: Experiences, Challenges, and Perspectives” aimed to assess the current state of AMR in the Eastern Mediterranean, discussing both country-specific and global data on the spread of resistance, and understanding its driving factors. It also aimed to raise awareness among professionals and the public about AMR-related issues and to share the latest knowledge on combating AMR across public health, animal health, and environmental health sectors.

## Roundtable description

The roundtable session began with an in-depth presentation on antimicrobial resistance, highlighting global and regional challenges, associated risks, and the burden on health systems. The discussion also covered initiatives focused on controlling and preventing the spread of antibiotic-resistant bacteria. Participants shared their experiences in establishing AMR surveillance systems, building information networks on antibiotic-resistant bacteria, and utilizing this information to promote rational antibiotic use. Additionally, the session explored the stages of implementing national plans for the responsible use of antibiotics and raising awareness among stakeholders. This comprehensive session lasted for 2 hours.

## Findings

### AMR in EMR: experiences, challenges and perspectives

As noted earlier, AMR’s projected global mortality and economic burden are substantial, with significant implications for health systems and economies worldwide. AMR poses a considerable threat not only to human health but also to global food security, livestock production, and the economy, with the potential cost of inaction reaching up to $100 trillion by 2050 (1). She emphasized the role of the AMR Multi-Stakeholder Partnership Platform, which brings together various stakeholders from different sectors, including health, agriculture, and the environment, to promote collaborative action (7). The platform aims to optimize the use of antimicrobials, reduce the incidence of infections, and develop a One Health approach that spans human, animal, and environmental health. It also underscores the need for global cooperation and action, guided by initiatives such as the WHO’s Global Action Plan on AMR, and stresses the importance of political commitment and sustainable funding to address this growing global health threat (8).

In the context of the Eastern Mediterranean Region (EMR), the challenges related to AMR are compounded by regional factors such as conflict, weak health systems, and limited access to effective antibiotics. These issues are further exacerbated by the lack of coordinated AMR surveillance, making it difficult to gauge the true extent of the problem. Recent evidence shows alarming trends in resistance levels across the EMR, particularly in conflict-affected countries. A 2025 study found that carbapenem-resistant *Acinetobacter* spp. exceeded 65%, while MRSA rates peaked at 70.09% in these settings, indicating a critical need for focused interventions in fragile contexts (9). Speakers and participants emphasized that addressing these challenges requires the adoption of the One Health approach, which calls for collaboration across human, animal, and environmental sectors to create a comprehensive response to AMR. The platform fosters multi-sectoral engagement, encouraging stakeholders to work together in addressing antimicrobial usage in livestock, which impacts the livelihood of over 1.5 billion people globally who depend on animal-sourced foods for nutrition and income.

The importance of political commitment in the EMR was also emphasized, with particular focus on the need for sustainable funding mechanisms to support National Action Plans (NAPs) based on the WHO Global Action Plan. The platform aims to facilitate information sharing, collaboration, and action across sectors, with a specific focus on promoting antimicrobial stewardship and improving infection prevention and control measures in healthcare facilities. Strengthening surveillance systems in the region, particularly for antibiotic usage and resistance patterns, was identified as a critical step toward containing AMR and preventing its further spread. The partnership between WHO, the Food and Agriculture Organization (FAO), WOAH, and the United Nations Environment Programme (UNEP) under the Quadripartite Joint Secretariat offers a framework for integrated efforts to address AMR at regional and global levels.

### Role of WHO Collaborating Center for Reference and Research on Bacterial Pathogens

It was noted that the role of WHO collaborating center (CC) for Reference and Research on Bacterial Pathogens is crucial in this regard. AMR poses an escalating threat, exacerbated by conflict and displacement in the region. Displacement leads to poor sanitation, lack of healthcare infrastructure, and the misuse of antibiotics, which all contribute to the rise of drug-resistant pathogens (10). The World Health Organization Collaborating Center (WHO CC) supports regional and international efforts through laboratory support, the development of rapid diagnostics, and the training of healthcare workers on pathogen detection. The center also plays a key role in the in-emergency response and surveillance of bacterial pathogens, exemplified by its efforts during the 2022 cholera outbreak in Lebanon, where it identified two strains linked to Pakistan and Yemen. It emphasized the importance of a One Health approach and regional collaboration in combating AMR. Surveillance, education, and the development of effective stewardship programs are necessary to address this growing threat.

### The role of laboratories in addressing antimicrobial resistance

The critical role that laboratories play in combating AMR was emphasized. It outlines the necessity of laboratory-based surveillance programs to detect resistance, track its spread, and inform treatment decisions. Laboratories provide valuable surveillance data, helping to generate antibiograms, detect outbreaks, and support antimicrobial stewardship. Challenges such as limited staff capacity, access to diagnostics, and the need for enhanced training and equipment are highlighted (11). Moreover, the panel shared insights into the importance of quality assurance in laboratories, including internal and external quality control measures, such as participation in External Quality Assurance Schemes (EQAS). Overall, the role of laboratories is pivotal in AMR surveillance, treatment guidance, and in developing and monitoring intervention strategies.

### Antimicrobial resistance and infection prevention and control in healthcare settings

Speakers discussed the crucial role of IPC in combating AMR, especially in healthcare settings. While anyone can acquire a resistant infection, vulnerable groups such as the older adult and those with underlying health conditions face higher risks. These risks are exacerbated in healthcare environments due to factors like environmental contamination, medical devices, and shared equipment. AMR organisms can spread through surfaces, healthcare workers, patients, and visitors, and asymptomatic carriers may unknowingly transmit resistant organisms, often undetected due to limited screening tests. Key strategies to combat AMR include preventing new infections by reducing healthcare-associated infections (HAIs), improving antimicrobial use by ensuring the right drug, dose, and duration to slow resistance, and halting the spread of resistant germs through effective infection control practices (12). Strong IPC programs, led by trained teams at national and facility levels, are essential. These programs must encompass core components like guidelines, education, and HAI surveillance. However, many countries, especially low- and middle-income nations, still lack fully operational IPC programs. To effectively address AMR, the implementation of WHO’s core IPC components is particularly critical in regions like the Eastern Mediterranean, where progress remains limited.

### Global and regional one health initiatives with focus on AMR

The importance of the One Health approach in addressing AMR was discussed. The One Health Joint Plan of Action (OHJPA) focuses on strengthening health systems, reducing risks from zoonotic diseases, enhancing food safety, and curbing AMR, with an emphasis on multisectoral coordination to tackle AMR and emerging health threats (13). The Regional One Health Operational Framework, developed based on reviews from EMR countries, provides strategies for governance, collaboration, and community engagement. It has been endorsed by the Regional Committee, urging Member States to institutionalize One Health and prioritize interventions for zoonotic diseases, AMR, and food safety. Countries like Jordan, Egypt, and Sudan have already developed national frameworks and operational plans, strengthening joint risk assessments and multisectoral coordination to manage zoonotic diseases and AMR. The misuse and overuse of antimicrobials in both human and animal health are major drivers of AMR in the region, which has some of the highest and fastest-growing rates of antimicrobial consumption and resistance globally. With estimates predicting 39 million deaths attributable to AMR between 2025 and 2050, equivalent to three deaths every minute, the need for a One Health approach is urgent. Speakers and participants called for improved governance, data sharing, and capacity building across sectors, with IPC and biosecurity in animal health being key to preventing AMR. There is growing political momentum in the region, with countries like Saudi Arabia and Oman hosting ministerial conferences on AMR. The call-to-action urges countries to coordinate governance structures, enhance data sharing, and advocate for stronger multisectoral collaboration, with WHO supporting the development and operationalization of national One Health plans to address zoonotic diseases, AMR, and food safety.

### Curbing AMR in animals

The role of the World Organization for Animal Health in combating AMR in the animal sector was underscored by speakers. The WOAH strategy on AMR focuses on several key objectives: improving awareness and understanding of AMR, enhancing surveillance and research, supporting governance and capacity building, and promoting the implementation of international standards related to AMR. To raise awareness, WOAH utilizes social media, radio, and other platforms to disseminate AMR-related materials across over 100 countries, working with regional offices to tailor these materials to local contexts. In 2015, WOAH launched a global database to track antimicrobial agents used in animals, which later evolved into the ANIMUSE online system. This tool monitors antimicrobial usage (AMU) trends and helps enforce international codes regarding AMR. WOAH supports good governance by promoting the One Health approach, integrating animal health into broader AMR strategies, and assisting countries in developing NAPs. It has revised its standards on AMR to include companion animals and environmental protections, and advocates for reducing the use of antimicrobials in animals, particularly discouraging their use as growth promoters. Policy briefs from WOAH encourage responsible antimicrobial use, promote alternative treatments, and enhance biosecurity on farms to reduce AMR risks. Surveillance data from WOAH shows progress in reporting AMU among member countries, but some members still use antimicrobials as growth promoters or without proper risk analysis, failing to fully comply with international standards. The Muscat Manifesto aims to reduce antimicrobial usage in the agri-food system by 30–50% by 2030, with a goal of achieving zero use of medically important antimicrobials for non-veterinary purposes (14).

## Discussion

AMR is an evolving public health threat in EMR due to high levels of antimicrobial consumption, weak healthcare systems, and ongoing conflicts. AMR is responsible for millions of deaths globally, with the EMR contributing significantly due to the lack of coordinated response mechanisms. One study estimated that AMR-related mortality in the region is driven by the misuse of antibiotics, both in the healthcare and agriculture sectors, combined with unregulated access to these medications. Given the significant global mortality and economic impact outlined in the introduction, urgent regional action is warranted to prevent further escalation in the EMR. The economic burden of AMR is equally staggering, with projections of up to $100 trillion in global economic losses by 2050 if no action is taken. AMR impacts not only human health but also livestock, agriculture, and food security, which are critical in many EMR countries heavily dependent on agriculture. Key interventions, such as optimizing antimicrobial use, are urgently needed.

The silent nature of AMR, along with the misuse and overprescription of antibiotics, especially in low- and middle-income countries, is a significant factor in this growing threat. In response, the World Health Assembly passed Resolution 67.25 in 2014, requiring countries to develop and implement national action plans to combat AMR (15). Several countries in the EMR lack strict regulatory frameworks governing the sale and distribution of antibiotics, leading to easy availability without a prescription. This is exacerbated by insufficient public awareness about the dangers of misuse and the absence of standardized guidelines for antibiotic prescription among healthcare providers. Effective policies from other regions highlight the need for comprehensive stewardship programs, including robust prescription regulations and public health campaigns to educate both healthcare professionals and the public about the responsible use of antibiotics (16).

The AMR Multi-Stakeholder Partnership Platform serves as a vital initiative in fostering collaboration between various sectors, health, agriculture, and the environment under the One Health framework. This platform promotes good governance, antimicrobial stewardship, and improving IPC. According to the Global Action Plan on AMR, political commitment is essential for the region, with many countries yet to fully integrate AMR into their national health strategies. The AMR Multi-Stakeholder Partnership Platform is one of the major bodies fostering the sustainable development of evidence-based solutions to AMR (7). Leadership and stakeholder commitment at both national and regional levels are critical for the success of AMR and antimicrobial stewardship (ASP) initiatives. High-level engagement ensures alignment of priorities, facilitates intersectoral coordination, and secures needed resources. Regional summits and forums, such as the EMPHNET Conference, serve as important platforms to drive this commitment from top management and decision-makers. Digital media and education campaigns play a crucial role in raising awareness and influencing behavior change regarding AMR. These tools have proven effective in disseminating information quickly and broadly, reaching diverse populations, including those in remote and underserved areas. Campaigns such as the World Health Organization’s World Antibiotic Awareness Week use social media platforms to engage the public and promote responsible antibiotic use, helping to reduce the misuse that contributes to AMR. In addition to digital and community campaigns, integrating AMR education into school curricula across the EMR could offer a sustainable approach to behavior change. Countries can build a generation equipped with knowledge about antibiotic resistance, its risks, and the importance of stewardship by promoting responsible antimicrobial use from a young age.

Beyond awareness campaigns, sustainable and systemic change requires investment in the health workforce and educational systems. A train-the-trainer (ToT) approach targeting regional doctors, nurses, and healthcare workers can help scale up AMR/ASP knowledge and ensure consistent practices across facilities. At the same time, integrating AMR and stewardship content into undergraduate and postgraduate curricula for medical, nursing, and pharmacy students across the region would institutionalize this knowledge and support long-term behavior change. Additionally, greater engagement with pharmacists and regulatory enforcement to stop the over-the-counter sale of antibiotics, especially in low- and middle-income countries, remains a critical and often overlooked intervention.

For the EMR, further investment is necessary in building laboratory capacities, enhancing surveillance, and ensuring that health systems are equipped to handle resistant pathogens. Countries like Jordan and Egypt have developed NAPs on AMR, but regional disparities in resources, expertise, and political will present ongoing challenges. Additionally, the lack of dedicated funding for AMR and ASP programs at the institutional level remains a major barrier. Allocating specific budgets would enable hospitals and health facilities to establish dedicated AMR/ASP teams, conduct gap analyses, and ensure consistent data reporting to national surveillance systems.

Across the EMR, progress in implementing NAPs varies significantly. Jordan and Egypt have made notable progress in operationalizing their NAPs mainly through investing in laboratory infrastructure, antimicrobial stewardship programs, and multisectoral coordination mechanisms. Other countries in the EMR, like Saudi Arabia and Oman, have further demonstrated strong political momentum by hosting high-level ministerial conferences to support implementation (17, 18).

On the other hand, conflict-affected countries, such as Yemen, Syria, and Afghanistan, face systemic barriers that limit their progress. The literature indicates that these countries struggle with disrupted, fragile health systems and under-resourced laboratories. Afghanistan shows how fragile healthcare infrastructure, unregulated antibiotic access, and limited public awareness can exacerbate AMR, with reported resistance rates exceeding 80% among key pathogens and persistent challenges in surveillance and infection control (19). Such disparities in the region highlight the need for tailored strategies that build on the progress in stable settings while also directing targeted support toward fragile settings to strengthen governance, surveillance, and regulatory systems.

AMR is particularly prevalent in conflict zones, where disruptions to healthcare systems, sanitation, and infrastructure contribute to the emergence and spread of infections and resistance (20). For example, during the Syrian conflict, multi-drug-resistant strains of *Acinetobacter* emerged, spreading into Lebanon through wounded civilians (21). These strains are not only multidrug-resistant but also represent a significant threat to regional healthcare systems.

The WHO CC for Reference & Research on Bacterial Pathogens plays a vital role in addressing AMR in vulnerable populations affected by displacement. The displacement crises in countries like Syria, Yemen, and Lebanon have disrupted healthcare systems, leading to widespread misuse of antibiotics. Many refugees, lacking access to healthcare professionals, resort to over-the-counter antibiotics for common illnesses, which contributes to the development and spread of resistant bacteria. The WHO CC provides crucial laboratory support, ensuring that bacterial pathogens are accurately identified, resistance patterns are tracked, and healthcare workers are trained to manage outbreaks effectively. For example, during the 2022 cholera outbreak in Lebanon, WHO CC quickly identified the strains of cholera, linking them to sources in Pakistan and Yemen, and provided critical guidance on containment strategies. Through initiatives such as PulseNet Middle East and whole-genome sequencing, these efforts have enhanced the region’s ability to monitor and respond to AMR outbreaks (22). However, shortages of funds and poor infrastructure remain major challenges to efficient AMR surveillance in conflict-affected countries.

Laboratories are at the heart of any successful AMR strategy. Laboratory-based surveillance provides the data needed to inform national AMR policies, treatment guidelines, and antimicrobial stewardship programs. However, many EMR countries face significant obstacles in establishing well-functioning laboratory networks capable of detecting and tracking AMR. Critical need for laboratories to generate reliable antibiograms, which help clinicians make informed decisions about antibiotic use was also noted. These antibiograms are pivotal in guiding treatment decisions and preventing inappropriate antibiotic use. One challenge in the EMR is the limited capacity of laboratories to perform routine diagnostics due to inadequate infrastructure, training, and resources. Many laboratories in low-resource settings lack access to essential diagnostic tools, such as automated blood culture systems, which are key to identifying bacterial infections early and accurately.

Additionally, quality assurance is a significant concern, as many laboratories struggle to maintain internal and external quality control measures. Participation in EQAS is necessary to ensure that laboratory diagnostics are accurate and comparable across regions. Laboratories must also adopt best practices for biosafety and biosecurity, particularly when dealing with highly resistant pathogens. To improve AMR surveillance and control, it is essential to build laboratory capacities, provide ongoing training to laboratory staff, and strengthen laboratory networks across the EMR to ensure timely and accurate data for AMR monitoring.

IPC is a cornerstone of AMR mitigation, particularly in healthcare settings where antibiotic use is highest. Hospitals and clinics are often breeding grounds for resistant infections due to high antimicrobial use, invasive procedures, and close contact between patients. The panelists emphasized the disproportionate impact of AMR on vulnerable populations such as the older adult and immunocompromised. In healthcare settings, resistant organisms can spread rapidly through medical devices, contaminated surfaces, and direct contact between healthcare workers and patients. HAIs, particularly those caused by resistant organisms like methicillin-resistant *Staphylococcus aureus* (MRSA) or multidrug-resistant *Acinetobacter baumannii*, pose significant threats.

IPC strategies are essential to prevent the spread of resistant infections. Effective IPC programs should include robust hand hygiene, environmental cleaning, and patient screening to detect carriers of resistant organisms. Unfortunately, many countries in the EMR lack fully functional IPC programs, and even when guidelines exist, implementation is often inconsistent. WHO’s core components for IPC programs, including education, surveillance, and monitoring, are crucial for hospitals to reduce the spread of AMR. However, in low-resource settings, these programs are often underfunded and understaffed, resulting in higher rates of resistant infections.

OHJPA calls for stronger health systems, improved governance, and enhanced collaboration across sectors to address zoonotic diseases, food safety, and AMR. For example, reducing antimicrobial use in agriculture is a critical component, as overuse in livestock is a major driver of resistance. Countries like Jordan and Sudan are developing frameworks that focus on joint risk assessments and multisectoral data sharing to manage these threats effectively. Political momentum in the region is building, with countries like Saudi Arabia hosting ministerial conferences on AMR and working with international organizations to build stronger health systems. However, further work is needed to fully operationalize the One Health approach, particularly in terms of improving data sharing between sectors and ensuring that AMR is integrated into national policies for both human and animal health.

Addressing AMR in the animal sector is essential, as antimicrobial use in livestock, especially for growth promotion, is a major driver of resistance. WOAH plays a pivotal role in promoting responsible antimicrobial use and supporting countries in developing national policies. WOAH’s ANIMUSE system, which tracks antimicrobial use in animals, has been instrumental in helping countries monitor trends and enforce international standards. However, compliance with these standards varies, with some countries still allowing antimicrobials to be used as growth promoters or without proper risk assessments. WOAH advocates for reducing antimicrobial use in animals, particularly for non-therapeutic purposes. It promotes alternative practices such as improved farm hygiene, biosecurity, and the use of vaccines to prevent infections in livestock, thereby reducing the need for antibiotics. Biosecurity measures on farms, such as quarantine practices and protective barriers, are essential to prevent the spread of infections between animals and humans.

The Muscat Manifesto and upcoming ministerial conferences on AMR will be key platforms for strengthening global and regional efforts to reduce antimicrobial use in agriculture and prevent the spread of resistant organisms across sectors. The 2016 UN General Assembly declaration on AMR laid the foundation for global action. However, more concrete targets and cross-sectoral collaboration are required to meet the growing challenge. The 2024 UN High-Level Meeting on AMR provides an opportunity to accelerate efforts (23), focusing on investing in present-day interventions and securing long-term solutions.

## Limitations

This article is based on discussions and presentations from a dedicated roundtable session at EMPHNET’s Eighth Regional Conference. Thus, the findings in this paper reflect the perspectives and experiences of participating experts and may not represent a comprehensive or systematic review of all available evidence on AMR in the EMR. The analysis is limited by the absence of primary data collection and reliance on existing literature and expert opinion shared during the roundtable. These limitations should be considered when interpreting the findings and recommendations presented.

## Conclusion

AMR is a complex and evolving threat in the EMR due to multiple factors, including high antimicrobial consumption, inadequate healthcare infrastructure, conflict-induced displacement, and weak regulatory frameworks. AMR is already responsible for significant mortality worldwide, with severe economic consequences. The EMR faces unique challenges, such as unregulated antibiotic sales, insufficient public awareness, and the effects of conflict on healthcare systems.

Despite these challenges, global initiatives like the AMR Multi-Stakeholder Partnership Platform and WHO Collaborating Centers are fostering cross-sectoral collaboration under the One Health framework, offering a coordinated response to AMR. Several countries in the EMR have advanced their NAPs through investments in laboratory capacity, antimicrobial stewardship, and multisectoral coordination while others, particularly conflict-affected states, continue to face significant barriers.

### Recommendations

To address the growing threat of AMR in the EMR and build on the progress achieved to date, the following priority actions are proposed. These recommendations aim to strengthen governance, enhance technical capacity, and promote sustainable, multisectoral solutions that can be adapted to the diverse contexts across the region.

1. Strengthen regulatory frameworks for antimicrobial use: Countries in the EMR should develop and enforce stricter regulations governing the sale and distribution of antibiotics, ensuring that they are only available by prescription. This includes revising national policies to incorporate comprehensive antimicrobial stewardship programs, ensuring facilities receive adequate funding to build teams, conduct gap analyses, and contribute data to national AMR systems.
2. Enhance laboratory capacities and surveillance: Investing in laboratory infrastructure and training is critical for effective AMR surveillance. Expanding participation in EQAS and adopting new diagnostic technologies, such as whole-genome sequencing, can improve the accuracy and timeliness of AMR detection and outbreak response.
3. Promote public awareness and education campaigns: Digital media and education campaigns targeting both healthcare professionals and the public can play a crucial role in promoting responsible antibiotic use. Initiatives like the WHO’s World Antibiotic Awareness Week should be expanded and tailored to local contexts to improve knowledge and reduce the misuse of antibiotics.
4. Adopt and strengthen the One Health approach: The One Health framework, which integrates human, animal, and environmental health, should be institutionalized across the EMR. This approach encourages collaboration between sectors to address zoonotic diseases, food safety, and antimicrobial resistance. Countries should prioritize multisectoral data sharing and joint risk assessments to manage these threats.
5. Implement and scale up infection prevention and control programs: Healthcare systems, particularly in low- and middle-income countries, need to scale up IPC programs to prevent the spread of resistant infections in hospitals and clinics. Implementing WHO’s core IPC components, such as hand hygiene, environmental cleaning, and patient screening, should be a priority to reduce healthcare-associated infections.

## Data Availability

The original contributions presented in the study are included in the article/supplementary material, further inquiries can be directed to the corresponding authors.
